# Impacts of *Macleaya cordata* on Productive Performance, Expression of Growth-Related Genes, Hematological, and Biochemical Parameters in Turkey

**DOI:** 10.3389/fvets.2022.873951

**Published:** 2022-07-11

**Authors:** Eman A. Manaa, Mervat A. Abdel-Latif, Samya E. Ibraheim, Abdelaziz Sakr, Mahmoud Dawood, Ghadeer M. Albadrani, Attalla F. El-kott, Mohamed M. Abdel-Daim, Basant M. Shafik

**Affiliations:** ^1^Animal and Poultry Production, Department of Animal Wealth Development, Faculty of Veterinary Medicine, Benha University, Benha, Egypt; ^2^Department of Nutrition and Veterinary Clinical Nutrition, Faculty of Veterinary Medicine, Damanhour University, Damanhour, Egypt; ^3^Rabbit, Turkey and Waterfowl Animal Production Research Institute, Agricultural Research Center, Giza, Egypt; ^4^Department of Biotechnology, Animal Production Research Institute, Agricultural Research Center, Giza, Egypt; ^5^Department of Animal Production, Faculty of Agriculture, Kafrelsheikh University, Kafr El-Sheikh, Egypt; ^6^Center for Applied Research on the Environment and Sustainability, American University in Cairo, New Cairo, Egypt; ^7^Department of Biology, College of Science, Princess Nourah bint Abdulrahman University, Riyadh, Saudi Arabia; ^8^Biology Department, Faculty of Science, King Khalid University, Abha, Saudi Arabia; ^9^Zoology Department, College of Science, Damanhour University, Damanhour, Egypt; ^10^Department of Pharmaceutical Sciences, Pharmacy Program, Batterjee Medical College, Jeddah, Saudi Arabia; ^11^Pharmacology Department, Faculty of Veterinary Medicine, Suez Canal University, Ismailia, Egypt

**Keywords:** *Macleaya cordata*, growth performance, gene expression, Sangrovit^®^, Turkey

## Abstract

*Macleaya cordata (M. cordata)* is a herbal plant that has abundant amounts of sanguinarine, which has many biomedical properties. The effects of *M. cordata* dietary supplementation on the productive performance, some blood constituents, and growth-related genes' expression were evaluated in turkey. *M. cordata* extract was dietary supplemented to turkey at levels of 25, 50, and 100 ppm and a control group. Growth performance measurements (FBW, ADG, and FCR) and production efficiency factor for turkey (BPEF) were similar (*p* > 0.05) in all supplemented groups. *M. cordata* has no adverse effects (*p* > 0.05) on the birds' health regarding hematological (Hb, RBCs, WBCs, and PCV) and blood biochemical indices evaluating liver function, kidney function, and lipid profile. Moreover, the mRNA expression of growth-related genes, such as growth hormone receptor (GHR), insulin-like growth factor 1 (IGF-1), cyclooxygenase 3 (COX-3), adenine nucleotide translocase (ANT), and uncoupling protein 3 (UCP-3) were upregulated (*p* < 0.001) in *M. cordata* treatments with the highest value for SG50 compared with the control group. We concluded that exogenous *M. cordata* dietary supplementation upregulated the expression of growth-related genes in turkey at a level of 50 ppm without adverse effects on their health status regarding hematological and biochemical indices.

## Introduction

The increasing population and food demand require more food production and sustainable plans to ensure food availability for the next generations. In the poultry industry, the probability of infectious diseases is high, resulting in a substantial economic loss and antibiotic resistance and other environmental hazards. Under the United Nations and Food and Agriculture Organization regulations involved in food security and the production of safe animal protein products, it has become necessary to develop natural additives to substitute antibiotic usage. The supplementation of phytogenic feed additives is proven safe and efficient to be widely applied in fish, birds, and livestock production for their productive efficiency if used in the optimal dose and reasonable duration (1, 2).

*Macleaya cordata* is a herbal plant that has abundant amounts of sanguinarine, which is commercially known as Sangrovit^®^ (3). The *M. cordata* plant contained in Sangrovit^®^ (as a feed additive used in animal nutrition) is reliable as per the list of the European Food Safety Authority (4). Furthermore, Sangrovit^®^ is not metabolized into potentially harmful metabolites and is excreted without being absorbed through the small intestine (3). Traditionally, *M. cordata* is used as a Chinese herbal with many biomedical properties (depurative, analgesic, carminative, antiedemic, and diuretic) (3, 5). It contains alkaloid substances (e.g., benzophenanthidrine), which are extracted from the rhizomes of the *Sanguinaria canadensis* plant (6). Incorporating *M. cordata* in birds' diet resulted in improving the feed efficiency resulting from enhanced intestinal health (7–12). *M. cordata* has antioxidant activity as well as enhanced immunity in livestock and birds (13).

Turkey meat is preferred by consumers as a source of protein and essential amino and fatty acids, minerals, and vitamins. Recently, expensive raw materials made it imperative to search for alternative feed ingredients and feed additives that will help to reduce the overall cost of the rations. As a result, the application of phytobiotics besides understanding their potential effect on performance and wellbeing is crucial to protect against antibiotic resistance. To the best of the author's knowledge, there are no previous trials that have investigated the effects of *M. cordata* dietary inclusion in turkey. Therefore, the present study aimed to evaluate the impact of *M. cordata* dietary supplementation on the growth measurements, hematological, and biochemical parameters, and the expression of growth-related genes in turkey.

## Materials and Methods

### Ethics Statement

This investigation was approved by the Committee of Local Experimental Animal Care, Damanhour University, Egypt, Faculty of Veterinary Medicine (VMD: 15/2018).

### Birds and Dietary Treatments

One hundred and eighty one-day-old-Bronze turkey chicks were distributed into four experimental treatments of mixed-sex, randomly with 45 chicks in each. Each treatment was divided into 3 replicates (15 chick/replicate). The chicks were wing-banded for their identification. All chicks were maintained for 2 weeks for brooding with feeding on control (basal diet), for acclimatization at the beginning of the experiment, and the chicks were reared on a litter floor over a 20-week duration. Feeding and watering were freely accessed. All birds were reared under standard conditions. Chicks were exposed to 24-h light throughout the whole experimental period.

Birds were fed on a well-balanced control diet (basal diet) during the first 2 weeks, then, chicks were accessed to the experimental diets over the whole experiment (20^th^ week of age). The four groups (control, **SG25**, **SG50**, and **SG100**) were treated with 0, 25, 50, and 100 ppm Sangrovit^®^ (Germany, Registered Number: 816) and its composition was *M. cordata* extract 4% and powder *M. cordata* (carrier) 96%, with chemical analysis being crude protein 7%, crude fiber 7.2, crude ash 58.4, and moisture 4.0% (14). The basal diets (corn-soybean-based diet) were formulated according to the nutrient requirements for Bronze Turkey (Table 1).

**Table 1:** Ingredients and the composition of brooding and growing diets (%, as- fed basis).

**Items**	**Brooding (2–8 week)**	**Growing (8–20 week)**
**Ingredient (%)**
Yellow Corn	50.0	69.0
Soybean meal (44% Crude Protein)	39	20
Fish meal (64% Crude Protein)	10	10
Di-Calcium Phosphate	—	0.10
Ground limestone	0.40	0.30
D-L methionine	—	0.10
L-Lysine	0.10	0.15
Premix^*^	0.25	0.10
Salt (Sodium chloride)	0.25	0.25
**Chemical composition (calculated)**
^a^CP, %	27.5	20.87
^b^ME (kcal/kg)	2,830	3,000
^c^CF, %	3.9	2.99
^d^Ca, %	0.79	0.72
^e^Avail. P, %	0.43	0.42
^f^Lys., %	1.8	1.38
^g^Meth., %	0.78	0.8
^h^Meth.+ Cyst., %	0.9	0.82

a *Crude protein*;

b *metabolizable energy*;

c *crude fiber*;

d *calcium*;

e *available phosphorus*;

f *lysine*;

g *methionine*;

h *methionine, and cystein*.

* *Each 3 kg of premix contains the vitamin premix and trace mineral. The vitamin premix contributed the following: vitamin A 12,000,000 IU, vitamin D3 2,200,000 IU, vitamin E 10,000 mg, vitamin K3 2,000 mg, vitamin B1 1,000 mg, vitamin B2 4,000 mg, vitamin B6 1,000 mg, vitamin B12 10 mg, niacin 20,000 mg, biotin 50 mg, folic acid 1,000 mg, pantothenic acid 10,000 mg. The trace mineral contributed the following: copper sulfate 1,0000 mg, potassium iodide 1,000 mg, manganese oxide 55,000 mg, zinc oxide 50,000 mg, and selenium 100 mg*.

Turkey chicks were vaccinated in all cages as follows: Hitchner 1 in water (Polimun-ND^®^, BioTestLab, Kiev, Ukraine) at 7 days of age. Inactivated avian influenza subtype H5N1 vaccine (MeFluvac^®^, MEVAC, Cairo, Egypt) at 10 and 30 days of age; live Newcastle disease (Nobilis^®^ ND LaSota, MSD, Boxmeer, the Netherlands) vaccine in water at 16 days; inactivated Newcastle disease (MEVAC-ND^®^, MEVAC, Cairo, Egypt) at 45 days; inactivated bivalent (H5N1+ND) vaccine (Volvac^®^, Boehringer Ingelheim, Germany) at the age of 90 days and finally vaccinated with inactivated Cholera vaccine (Servac^®^, Abbasia, Egypt). The subcutaneous injection was used to administer all inactivated vaccines.

#### Growth Performance

Turkey chicks were weighed at 1 day old and then every month for 20 weeks. Bodyweight gain (**BWG**), relative growth rate (**RGR**), average feed intake (**AFI**), feed conversion ratio (**FCR**), and production efficiency factor for turkey (**BPEF**) (15) were evaluated throughout the whole experimental period.

#### Sample Collection

Blood samples (*n* = 16) were collected from the wing vein (2 ml) at the ages of 2^nd^ and 20^th^ weeks. About 1 ml of blood was collected on ethylenediaminetetraacetic acid (**EDTA**) to assess the blood hematology and 1 ml was centrifuged at 3,500 rpm/15 min and the extracted plasma was used for all biochemical analyses using commercial kits. Birds were euthanized using sodium pentobarbital (I/V; 50 mg/kg), immediately necropsied, and liver samples of 30 mg (*n* = 16; 8 males; and 8 females) were collected from each group and kept at −80°C for messenger RNA (mRNA) gene expressions.

#### Hematological and Biochemical Parameters

Biochemical parameters [liver function; glutamic oxaloacetic transaminase **(GOT)** and glutamic pyruvic transaminase **(GPT)**, kidney function; urea, creatinine, and blood urea nitrogen **(BUN)**, lipid profile; triglycerides, cholesterol, high-density lipoprotein **(HDL)**, and low-density lipoprotein **(LDL)**] were analyzed using commercial kits (Spinreact, Barcelona, Spain) on an ultraviolet-visible spectrophotometer. Blood indices, such as white blood cells (**WBCs**) and red blood cells (**RBCs**) were counted in a Neubauer hemocytometer using a 1:200 dilution with Natt and Herrick solution. Differential leukocyte count, hemoglobin (**Hb**) concentration, and packed cell volume (**PCV**) were determined as described previously (16).

#### Gene Expression

Following the manufacturer's protocol, total RNA was extracted from the tissue by TRIzol reagent (Invitrogen/Life Technologies, Carlsbad, CA, USA) and NanoDrop for quantification was utilized for RNA extraction. Single-stranded cDNA was synthesized from 1,000 ng of total RNA according to the manufacturer's High-Capacity cDNA Reverse Transcription Kits (Applied Biosystems) protocol. Cycling conditions were as follows: 25°C for 10 min, 37°C for 120 min, and 85°C for 5 min. Then total RNA and cDNA samples were stored at −80°C till the next step. The real-time polymerase chain reaction (RT-PCR) was used to evaluate growth hormone receptor (**GHR**), insulin-like growth factor 1 (**IGF-1**), cyclooxygenase 3 (**COX-3**), adenine nucleotide translocase (**ANT**), and uncoupling protein 3 (**UCP-3**) expression. RT-PCR assays were performed using the fluorescent dye SYBR Green (SYBR^®^ Green PCR Master Mix, Applied Biosystems, USA). All reactions were analyzed and normalized to the ROX Reference Dye (Invitrogen, Carlsbad, CA, USA). Amplification reactions were carried out referring to Gene Bank (http://www.ncbi.nlm.nih.gov) and β-actin was used as an endogenous control gene (Table 2). Gene expression was calculated from the obtained cycle threshold (Ct) values provided by RT-PCR instrumentation using the comparative Ct method to a reference (housekeeping) gene (β-actin), fold change = 2^−ΔΔCT^ as previously stated (23).

**Table 2:** Primers of candidate gene used in gene expression analysis.

**Target genes**		**Primer sequence (5**^′^**-3^**′**^)**	**Annealing temperature (**°**C)**	**Amplicon (bp)**	**Accession number and References**
GHR	Forward	AACACAGATACCCAACAGCC	60°C	145	KF957983.1
	Reverse	AGAAGTCAGTGTTTGTCAGGG			(17)
IGF-I	Forward	CACCTAAATCTGCACGCT	60°C	140	NM_001004384.2
	Reverse	CTTGTGGATGGCATGATCT			(18)
ANT	Forward	TGTGGCTGGTGTGGTTTCCTA	60°C	67	AB088686.1
	Reverse	GCGTCCTGACTGCATCATCA			(19)
UCP-3	Forward	GCAGCGGCAGATGAGCTT	60°C	62	XM_015280964.2
	Reverse	AGAGCTGCTTCACAGAGTCGTAGA			(20)
COX-3	Forward	AGGATTCTATTTCACAGCCCTACAAG	60°C	71	KC847746.1
	Reverse	AGACGCTGTCAGCGATTGAGA			(21)
β-actin	Forward	ACCCCAAAGCCAACAGA	60°C	136	NM_205518.1
	Reverse	CCAGAGTCCATCACAATACC			(22)

#### Statistical Analysis

The Shapiro–Wilks test was used to examine the normal distribution of variables. The statistical software package SPSS 20 (IBM 20; SPSS Inc., Chicago, IL, USA) was used based on the analysis of variance (ANOVA). Linear and quadratic contrasts of Sangrovit^®^ dietary inclusion were used to evaluate its effect on different measurements (growth, hematological, and biochemical parameters). Graphpad prism 5 was used to analyze the data of the RT-PCR with one-way ANOVA. Statistical significance between mean values and Tukey's *post-hoc* test was assessed at *p* < 0.05.

## Results

The effects of the experimental diets on performance traits in turkeys throughout the experiment are presented in Table 3. The results of linear contrast showed that growth was unaffected by Sangrovit^®^ inclusion, however, it was marginally improved (*p* > 0.05) in SG50 over the whole experimental period compared with control, by 6.29, 6.6, 5.6, and 14.9% for FBW, ADG, FCR, and BPEF, respectively.

**Table 3:** Effect of *Macleaya cordata* dietary supplementation on growth performance of turkey.

**Items**	**Control**	**Sangrovit supplementation**			***p*** **-value for Sangrovit**		
		**SG25**	**SG50**	**SG100**	**Control vs. others**	^g^**Lin**.	^h^**Quad**.
Initial weight, g	268.60 ± 3.64	268.50 ± 2.92	269.40 ± 3.28	268.60 ± 2.42	0.99	0.95	0.91
^a^FBW, g	4859.40 ± 133.02	5045.80 ± 146.4	5165.00 ± 148.13	4949.00 ± 126.9	0.44	0.52	0.14
^b^ADG, g	32.79 ± 0.93	34.12 ± 1.04	34.97 ± 1.05	33.44 ± 0.90	0.43	0.51	0.13
^c^RGR, %	178.50 ± 0.48	179.14 ± 0.54	179.59 ± 0.48	178.97 ± 0.47	0.48	0.39	0.19
^d^FI, g	16727.19 ± 221.31	16645.0 ± 206.03	16683.0 ± 221.31	16707.0 ± 224.89	0.99	0.98	0.81
^e^FCR	3.77 ± 0.11	3.63 ± 0.12	3.56 ± 0.12	3.68 ± 0.10	0.6	0.47	0.25
^f^BPEF	138.46 ± 7.40	151.59 ± 8.85	159.13 ± 9.32	144.00 ± 7.16	0.31	0.5	0.08

*values (means ± SE) within the same row carrying different superscripts are significantly different (p ≤ 0.05)*.

a *Final body weight*,

b *Average daily gain*,

c *Relative growth rate*,

d *Feed intake*,

e *Feed conversion ratio*,

f *production efficiency factor for turkey*,

g *Linear, and*

h *Quadratic*.

Contrast analyses of hematological and biochemical parameters are displayed in Tables 4, 5 during the experimental period at the age of 2^nd^ and 20^th^ weeks, respectively. No meaningful changes were recorded among the birds' groups that delivered *M. cordata* in terms of hematological and blood biochemical traits at the age of 2^nd^ week, except for white blood cell (WBC), SG50 recorded the highest value (*p* < 0.05) compared with other supplemented groups, and triglyceride, SG50 showed the least value (*p* < 0.05) compared with SG100. Similarly, at 20^th^ week among the birds' groups that delivered *M. cordata*, the changes recorded for mean cell volume (MCV) where SG50 was the highest value (*p* < 0.05) compared with SG25, WBC, SG50 recorded the highest value (*p* < 0.05) compared with control and other supplemented groups, GOT where SG100 showed the highest value (*p* < 0.05) compared with control, triglyceride where SG50 and SG100 recorded the least value (*p* < 0.05) compared with SG25, HDL where SG100 showed the highest value (*p* < 0.05) compared with SG25.

**Table 4:** Effect of *Macleaya cordata* dietary supplementation on hematological and biochemical parameters of turkey at the 2^nd^ week of age.

**Items**	**Control**	**Sangrovit supplementation**			***p*** **-value for Sangrovit**		
		**SG25**	**SG50**	**SG100**	**Control vs. others**	**Lin**.	**Quad**.
Hb (g/dl)	18.19 ± 0.59	19.05 ± 0.20	18.31 ± 0.56	19.18 ± 0.66	0.45	0.36	1
HCT (%)	54.56 ± 1.78	57.15 ± 0.61	54.94 ± 1.69	57.53 ± 1.97	0.45	0.36	1
RBC (Cells/ul)	6.06 ± 0.20	6.35 ± .0.07	6.09 ± 0.19	5.97 ± 0.43	0.74	0.64	0.43
MCV (fl/cell)	90.06 ± 0.01	90.04 ± 0.01	90.00 ± 0.00	90.28 ± 0.17	0.13	0.13	0.09
MCH (pg/cell)	30.00 ± 0.00	30.00 ± 0.00	30.00 ± 0.00	30.03 ± 0.03	0.4	0.19	0.33
MCHC (g/dl)	33.30 ± 0.00	33.30 ± 0.00	33.30 ± 0.00	33.24 ± 0.06	0.4	0.19	0.33
PLT (× 10^3^/mm3)	2787.50 ± 304.10	1993.80 ± 162.68	2487.50 ± 219.2	2300.00 ± 228.73	0.13	0.91	0.22
WBC (× 10^3^/mm3)	1275.00 ± 75.00^ab^	850.00 ± 82.38^b^	1806.20 ± 33.77^a^	925.00 ± 112.99^b^	0.01	0.36	0.21
GOT (U/L)	273.75 ± 15.92	277.00 ± 12.99	275.00 ± 10.90	288.00 ± 15.07	0.87	0.51	0.72
GPT (U/L)	8.00 ± 0.71	8.00 ± 0.85	9.75 ± 1.24	10.38 ± 2.64	0.61	0.21	0.84
Creatinine (mg/dl)	0.20 ± 0.03	0.19 ± 0.02	0.18 ± 0.02	0.20 ± 0.00	0.76	0.89	0.34
Urea (mg/dl)	6.55 ± 0.39	7.34 ± 0.52	7.45 ± 0.38	7.73 ± 0.30	0.22	0.06	0.54
BUN (mg/dl)	3.06 ± 0.18	3.42 ± 0.24	3.47 ± 0.18	3.60 ± 0.14	0.22	0.06	0.54
Triglyceride (mg/dl)	27.75 ± 5.52^ab^	31.38 ± 10.20^ab^	16.38 ± 2.35^b^	52.25 ± 12.14^a^	0.04	0.13	0.07
Cholesterol (mg/dl)	112.25 ± 8.72	127.62 ± 7.36	125.25 ± 8.88	124.12 ± 11.02	0.63	0.42	0.37
HDL (mg/dl)	79.63 ± 7.18	75.88 ± 5.42	73.63 ± 3.04	77.75 ± 3.84	0.85	0.73	0.45
LDL (mg/dl)	35.29 ± 3.41	45.58 ± 4.04	48.25 ± 8.65	41.09 ± 9.12	0.57	0.45	0.94

*Values (means ± SE) within the same row carrying different superscripts are significantly different (p ≤ 0.05). Hb, hemoglobin; HCT, Hematocrit; RBC, Red Blood Cells; MCV, Mean Cell Volume; MCH, Mean Cell Hemoglobin; MCHC, Mean Corpuscular Hemoglobin Concentration; PLT, Platelet; WBC, White Blood Cell; GOT, Glutamic Oxaloacetic Transaminase; GPT, Glutamic Pyruvic Transaminase; BUN, Blood Urea Nitrogen; HDL, High-Density Lipoprotein; LDL, Low-Density Lipoprotein; Lin., Linear; Quad., Quadratic*.

**Table 5:** Effect of *Macleaya cordata* dietary supplementation on hematological and biochemical parameters of turkey at the 20^*th*^ week of age.

**Items**	**Control**	**Sangrovit supplementation**			***p*** **-value for Sangrovit**		
		**SG25**	**SG50**	**SG100**	**Control vs. others**	**Lin**.	**Quad**.
Hb (g/dl)	14.25 ± 0.82	15.34 ± 0.36	13.87 ± 0.78	14.50 ± 0.80	0.52	0.82	0.76
HCT (%)	42.75 ± 2.46	46.01 ± 1.07	41.59 ± 2.33	40.29 ± 3.57	0.43	0.3	0.37
RBC (Cells/ul)	4.75 ± 0.27	5.11 ± .0.12	4.61 ± 0.26	4.47 ± 0.40	0.42	0.31	0.37
MCV (fl/cell)	90.09 ± 0.02^ab^	90.02 ± 0.01^b^	90.11 ± 0.23^a^	90.08 ± 0.02^ab^	0.03	0.58	0.53
MCH (pg/cell)	30.01 ± 0.01	30.02 ± 0.00	30.03 ± 0.02	30.20 ± 0.00	0.27	0.79	0.55
MCHC (g/dl)	33.30 ± 0.00	33.30 ± 0.00	33.30 ± 0.00	33.30 ± 0.00	1	1	1
PLT (× 10^3^/mm3)	2406.20 ± 192.82	2677.50 ± 170.36	2700.00 ± 179.53	2800.00 ± 127.82	0.41	0.12	0.62
WBC (× 10^3^/mm3)	1060.00 ± 225.26^b^	1227.50 ± 267.07^b^	1950.00 ± 199.10^a^	1250.00 ± 129.56^b^	0.02	0.18	0.05
GOT (U/L)	288.50 ± 11.91^b^	316.75 ± 8.31^ab^	326.25 ± 6.78^a^	329.88 ± 12.84^a^	0.03	0.01	0.24
GPT (U/L)	10.00 ± 3.62	6.50 ± 0.57	7.12 ± 0.67	6.38 ± 0.53	0.49	0.23	0.47
Creatinine (mg/dl)	0.19 ± 0.01	0.18 ± 0.03	0.21 ± 0.03	0.21 ± 0.01	0.59	0.29	0.79
Urea (mg/dl)	11.02 ± 0.54	11.88 ± 0.16	12.43 ± 0.42	12.01 ± 0.20	0.06	0.03	0.12
BUN (mg/dl)	5.15 ± 0.25	5.55 ± 0.08	5.80 ± 0.20	5.65 ± 0.10	0.06	0.03	0.12
Triglyceride (mg/dl)	59.88 ± 8.43^ab^	76.50 ± 7.26^a^	52.50 ± 4.02^b^	49.88 ± 8.93^b^	0.04	0.09	0.17
Cholesterol (mg/dl)	146.00 ± 5.16	144.12 ± 18.01	125.38 ± 3.13	136.25 ± 1.60	0.42	0.27	0.51
HDL (mg/dl)	76.25 ± 3.63^a^	63.25 ± 4.01^b^	68.00 ± 3.54^ab^	75.63 ± 3.60^a^	0.05	0.86	0.01
LDL (mg/dl)	57.78 ± 5.10	65.58 ± 14.50	46.88 ± 5.30	50.65 ± 3.40	0.41	0.29	0.81

*Values (means ± SE) within the same row carrying different superscripts are significantly different (p ≤ 0.05). Hb, hemoglobin; HCT, Hematocrit; RBC, Red Blood Cells; MCV, Mean Cell Volume; MCH, Mean Cell Hemoglobin; MCHC, Mean Corpuscular Hemoglobin Concentration; PLT, Platelet; WBC, White Blood Cell; GOT, Glutamic Oxaloacetic Transaminase; GPT, Glutamic Pyruvic Transaminase; BUN, Blood Urea Nitrogen; HDL, High-Density Lipoprotein; LDL, Low-Density Lipoprotein; Lin., Linear; Quad., Quadratic*.

**Figure 1:**
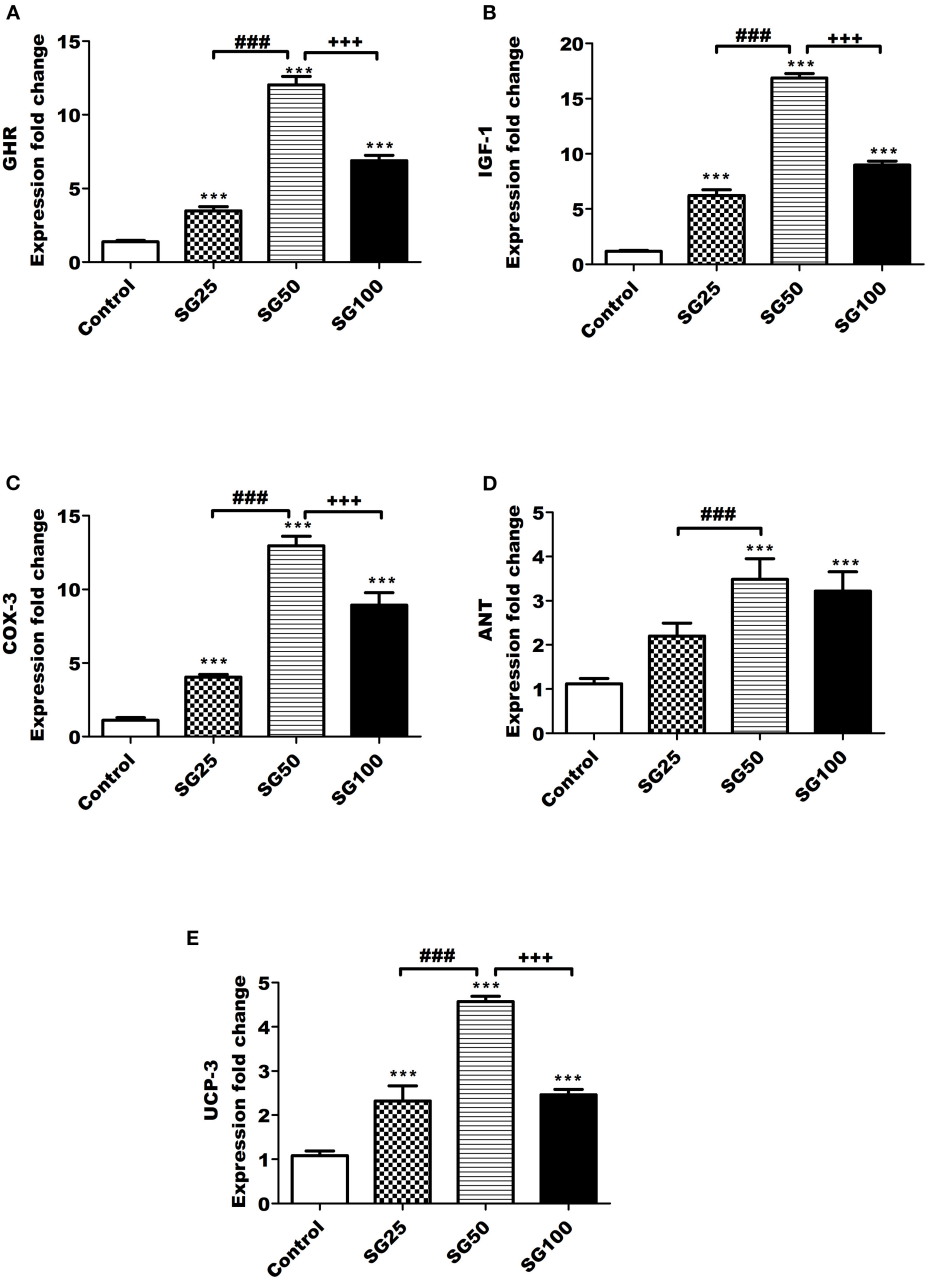
Real-time PCR **(**RT-PCR) validation of the growth hormone receptor *(GHR)* **(A)**, insulin-like growth factor 1 (*IGF-1*) **(B)**, cyclooxygenase 3 (*COX-3)* **(C)**, adenine nucleotide translocase adenine nucleotide translocase *(ANT)* **(D)**, and uncoupling protein 3 (*UCP-3)* **(E)** genes. ***p < 0.001 vs. control. ^###^*p* < 0.001 vs. SG25. ^+++^*p* < 0.001 vs. SG100, SG25, birds fed 25 ppm. SG50, birds fed 50 ppm. SG100, birds fed 100 ppm Sangrovit^®^.

The results of growth gene expression are shown in Figures 1A–E. Figures 1A–C showed that the expressions of GHR, insulin-like growth factor 1 IGF-1, and COX-3 in the Sangrovit^®^ supplemented groups were increased (*p* < 0.001), compared with control one, and their values upregulated (*p* < 0.001) in SG50 followed by SG100 and SG25, respectively. Data in Figure 1D show the mRNA expression of adenine nucleotide translocase (ANT); the SG50 group had higher (*p* < 0.001) level than control and SG25 groups. Moreover, its value was similar to SG100 group. As shown in Figure 1E, the fold change of uncoupling protein 3 (UCP-3) in SG50 group was upregulated (*p* < 0.001), compared with other groups, while their values in SG25 and SG100 groups were similar.

## Discussion

Using antibiotics is virtually required for maintaining the performance and wellbeing of birds to resist the expected infectious diseases and stressors. However, the crucial need to reduce chemotherapies to keep food security guided the researchers' need to continue their efforts in finding more sustainable solutions for birds' production (24). *M. cordata*, with its abundant amounts of sanguinarine, is an effective growth promoter and anti-bacterial additive (5, 11, 12). Concurrently, this study assessed the potential effect of *M. cordata* on growth performance regarding some growth-related genes, hematological, and biochemical parameters of turkeys during hatching and grow-out stages. The results have clearly shown similar growth rate (*p* > 0.05) in turkeys delivered *M. cordata* during hatching and grow-out stages. These findings are similar to Kozlowski et al. (25) who stated that *M. cordata* supplementation 20 ppm in the diet had no significant effect on FI, BWG, or FCR. The recommended dietary level of Sangrovit^®^ for broilers and growing turkeys was previously reported at 20–50 ppm. As a result, Zdunczyk et al. (26) investigated the dietary inclusion of Sangrovit^®^ at 30 ppm, which did not support performance parameters and was ineffective in protein utilization. In addition, Juskiewicz et al. (27) found that the FCR in both the starter and grower period were unaffected by 15 ppm Sangrovit^®^ supplementation. Moreover, Sangrovit^®^ dietary supplementation at the level of 0.05 and 0.1% had no significant differences among treatments regarding FI, feed utilization rate, and small intestine morphology (28).

On the other hand, some studies approved the positive effect of *M. cordata* in improving the growth of chickens and livestock animals either at a level of 25–50 ppm (29) or 30 ppm (30). The enhanced feed efficiency and nutrients' digestibility are previously reported in various studies that correlated the improved feed utilization in birds treated with *M. cordata* at a level of 20 and 50 ppm (31). Variations between these studies may be attributed to the differences in the composition of the used diets, age, and sex of the birds, dose of *M. cordata*, plant extracts, and other bioactive constituents.

Evaluation of turkey's health status can be explained by testing the hematological and blood biochemical traits (32). It gives a precise diagnosis of the metabolic rates, levels of nutrients in the blood, hematic and anemic status, and immune and antioxidative responses. The present study results showed no meaningful changes (*p* > 0.05) among the birds' groups delivered *M. cordata* at the age of 2^nd^ week in terms of hematological and biochemical indices as urea and creatinine (33). The obtained results regarding hematological and biochemical indices at the age of 20^th^ week are in agreement with Gilani et al. (34) who examined the efficacy of organic acids and phytobiotics in poultry feed, observing significant increases in RBC and WBC counts, as well as an increase in PCV in broiler chickens. These results confirm that dietary *M. cordata* has no adverse effects on turkey's health status in hatching or grow-out stages. The outputs of the experiment are in accordance with previous studies (8, 35–37) that approved *M. cordata* in in pig and poultry feeding without impairing their health and immune status; consumption of *M. cordata* in the feed at up to 1,000 mg/kg had no adverse effect on blood plasma. In contrast, both (31, 38) approved the cholesterol lowering effect of *M. cordata*.

Interestingly, the results showed upregulated growth-related genes, such as GHR, IGF-1, ANT, COX-3, and UCP-3 were upregulated (*p* < 0.001) in *M. cordata* supplemented groups with the highest value for SG50 in birds treated with *M. cordata*. These genes were investigated as promising biomarkers for enhancing the growth performance of quail (39). Previous studies clarifying the impact of *M. cordata* on the expression of the growth-related genes were limited; however, the related anti-inflammatory gene (e.g., NF-kB) showed upregulated expression in birds delivered *M. cordata* (13). The upregulated NF-kB was probably linked with the growth-related genes (e.g., IGF-1) and regulated its expression as a health-involved gene (40). However, intensive research is needed to determine the possible influence of *M. cordata* in controlling growth related genes in turkey. Although not relevant to the present study; yet other phytogenic extracts have been confirmed to upregulate animals' growth-related genes (41). Under the current trial conditions, growth performance is not correlated with the turkey's upregulated expression of growth-related genes. This may be explained as the genotypic effect is expressed earlier than the phenotypic performance, which appears after transcription of DNA into RNA, which is measured in our study (gene expression), and then RNA is translated into proteins to exert its function on the phenotypic performance (42).

## Conclusion

In conclusion, exogenous *M. cordata* dietary supplementation upregulated the expression of growth-related genes in turkey at a level of 50 ppm without adverse effects on their heath status regarding hematological and biochemical indices. Further research is needed to investigate the possible influence of *M. cordata* on gut health and in controlling growth-related genes in turkey in addition to its evaluation as an antibiotic alternative.

## Data Availability Statement

The data presented in this study are available on request from the corresponding author.

## Ethics Statement

This investigation was approved by the Committee of Local Experimental Animal Care, Damanhour University, Egypt, Faculty of Veterinary Medicine (VMD: 15/2018).

## Author Contributions

EM and MA-D: conceptualization and supervision. EM, MA-L, and MA-D: data curation. GA and AE-k: funding acquisition. SI: investigation. EM, MA-D, MA-L, AS, AE-k, and BS: methodology. AS and BS: resources. GA, AS, SI, MA-L, and BS: software. EM, MA-D, SI, GA, AS, MD, AE-k, MA-L, and BS: writing—review and editing. All authors contributed to the article and approved the submitted version.

## Funding

This research was supported by Princess Nourah bint Abdulrahman University Researchers Supporting Project number (PNURSP2022R30), Princess Nourah bint Abdulrahman University, Riyadh, Saudi Arabia. This research was supported by King Khalid University, Grant number (R.G.P.2/35/43), Abha, Saudi Arabia.

## Conflict of Interest

The authors declare that the research was conducted in the absence of any commercial or financial relationships that could be construed as a potential conflict of interest.

## Publisher's Note

All claims expressed in this article are solely those of the authors and do not necessarily represent those of their affiliated organizations, or those of the publisher, the editors and the reviewers. Any product that may be evaluated in this article, or claim that may be made by its manufacturer, is not guaranteed or endorsed by the publisher.
